# Supplementary material for: The Pharmacokinetic and Pharmacodynamic Relationship of Clinically Used Antiseizure Medications in the Maximal Electroshock Seizure Model in Rodents
Source: Int J Mol Sci. 2025 Jul 22;26(15):7029. doi: 10.3390/ijms26157029 (PMC12346480; doi:10.3390/ijms26157029)
Supplement: Supplementary file 1 [file ijms-26-07029-s001.zip › ijms-3669804-supplementary.pdf]

Supplemental Table S1. Summary of effective plasma concentrations, plasma protein binding (PPB), and unbound plasma concentrations ($C_{p,u}$) for the ASMs tested in mice, rats and humans.

	**Species**	**Effective plasma concentration (μM)***	**PPB(% bound)****	**$C_{p,u}$ EC_{50} (μM)*****
CBZ	Mouse	13.8	52.4	7.04
	Rat	4.55	63.9	1.63
	Human	17-51	75.0	4.25-12.8
PHT	Mouse	27.6	80.3	5.37
	Rat	3.20	80.6	0.89
	Human	40-79	90.0	4.00-7.90
VPA	Mouse	1668	12.0	1638
	Rat	1306	66.5	647
	Human	346-693	90.0	34.6-69.3
LSM	Mouse	19.3	21.8	15.6
	Rat	24.9	22.9	22.2
	Human	10-40	13.4	8.66-34.6
CNB	Mouse	66.0	43.2	40.1
	Rat	15.9	46.1	8.93
	Human	89.3-170	60.0	35.7-68.0
RTG	Mouse	2.26	86.2	0.52
	Rat	1.46	85.1	0.20
	Human	1.98-3.99	80.0	0.40-0.80

Note: ASMs: antiseizure medications; CNB: cenobamate; CBZ: carbamazepine; $C_{p,u}$: unbound plasma concentrations; EC_{50}: half maximal effective concentration; LSM: lacosamide; PHT: phenytoin; RTG: retigabine; VPA: valproic acid.

*For mouse and rat, plasma EC_{50} from the MES assay at one time point after a single dose is shown. For human, literature-reported therapeutic plasma concentrations are shown [36-47].

**PPB values for rodents were obtained from Xenon Pharmaceuticals' in-house data, except for VPA and CNB in mouse, for which published data were used. For human, published data is shown [36-47].

*** $C_{p,u}$ values for rodents have been calculated based on MES EC_{50}. $C_{p,u}$ values for humans represent the lower and upper bounds of reported therapeutic plasma concentrations, adjusted for PPB, as reported in clinical pharmacokinetic studies [36-47].

Supplemental Table S2. Diagnostics and goodness-of-fit metrics from logistic regression used for EC_{50} estimation in MES models

	Species	Tissue	EC_{50} (μM)*	Tjur's R^2	Cox–Snell's R^2	Log-likelihood ratio (G^2)	P-value
CBZ	Mouse	Plasma	13.8	0.422	0.403	53.6	<0.0001
		Brain	32.5	0.374	0.346	44.1	<0.0001
	Rat	Plasma	4.55	0.342	0.308	29.5	<0.0001
		Brain	4.70	0.325	0.293	27.7	<0.0001
PHT	Mouse	Plasma	27.6	0.496	0.444	23.5	<0.0001
		Brain	30.6	0.571	0.515	29.0	<0.0001
	Rat	Plasma	3.20	0.307	0.267	14.6	<0.001
		Brain	2.66	0.379	0.328	19.1	<0.0001
VPA	Mouse	Plasma	1668	0.706	0.606	20.5	<0.0001
		Brain	630	0.625	0.537	16.9	<0.0001
	Rat	Plasma	1306	0.647	0.563	19.1	<0.0001
		Brain	307	0.526	0.470	14.6	<0.001
LSM	Mouse	Plasma	19.3	0.634	0.530	60.4	<0.0001
		Brain	14.8	0.650	0.529	60.2	<0.0001
	Rat	Plasma	24.9	0.592	0.518	17.5	<0.0001
		Brain	12.2	0.617	0.556	19.5	<0.0001
CNB	Mouse	Plasma	66.0	0.626	0.529	46.6	<0.0001
		Brain	24.9	0.575	0.505	43.6	<0.0001
	Rat	Plasma	15.9	0.630	0.570	20.3	<0.0001
		Brain	10.7	0.668	0.595	21.7	<0.0001
RTG	Mouse	Plasma	2.26	0.376	0.356	17.6	<0.0001
		Brain	3.58	0.616	0.504	28.0	<0.0001
	Rat	Plasma	1.46	0.092	0.092	2.32	n.s.
		Brain	3.01	0.100	0.102	2.58	n.s.

Note: CNB: cenobamate; CBZ: carbamazepine; EC_{50}: half maximal effective concentration; LSM: lacosamide; PHT: phenytoin; RTG: retigabine; VPA: valproic acid.

*Logistic models were fitted to individual seizure probability values (ranging from 0 to 1), rather than raw binary outcomes. EC_{50} values represent the inflection point of the fitted curve, and confidence intervals were not derived due to the nature of the curve-fitting approach.
